# Supplementary material for: Prevalence and Cost of Antipsychotic Prescribing, within the Context of Psycholeptic Prescribing, in the Irish Setting
Source: Healthcare (Basel). 2024 Jan 29;12(3):338. doi: 10.3390/healthcare12030338 (PMC10855477; doi:10.3390/healthcare12030338)
Supplement: Supplementary file 1 [file healthcare-12-00338-s001.zip › healthcare-2780497-supplementary.pdf]

Supplementary Table S1. Drug Payment Scheme Thresholds (April 2019 - March 2022)

**Drugs Payment Scheme Thresholds**	
**Dates when threshold applied**	**Threshold Limit**
1 April 2019 to 1 November 2020	€124
1 November 2020 to 1 January 2022	€114
1 January 2022 to 1 March 2022	€100
Since 1 March 2022	€80

Reference:

Citizensinformation.ie. Drugs Payment Scheme [Internet]. Citizensinformation.ie; [cited 2023 Feb 8]. Available from: https://www.citizensinformation.ie/en/health/drugs_and_medicines/drugs_payment_scheme.html

Supplementary Table S2. GMS data per month over 32 months (Jan 2020-Aug 2022) for total costs (€), costs per example drug (€), number of patients per example drug, and total number of prescription items, and number of prescription items per example drug.

Variable	Obs	Mean	Std. Dev.	Min	Max
Total Cost	32	€4436469.18	121404.35	4146865.39	4656163.24
Total Cost Paliperidone	32	€608034.06	26534.54	554079.47	676078.57
Total Cost Quetiapine	32	€553411.53	20463.12	494866.39	583840.08
Total Cost Olanzapine	32	€488929.26	12974.68	450696.25	513404.44
Total Cost Aripiprazole	32	€364749.51	15373.01	328806.57	394876.23
No. of patients Paliperidone	32	1209.38	131.54	734.50	1300.00
No. of patients Quetiapine	32	27342.88	3304.53	16198.50	30282.00
No. of patients Olanzapine	32	16711.69	1874.16	9682.50	17648.00
No. of patients Aripiprazole	32	5455.78	641.95	3193.50	6008.00
Total No. of Prescription items	32	282792.62	7597.73	267412.00	297606.00
No. of prescription items Paliperidone	32	1443.88	59.59	1310.00	1535.00
No. of prescription items Quetiapine	32	33908.59	1750.65	29507.00	36921.00
No. of prescription items Olanzapine	32	20431.16	543.10	18823.00	21346.00
No. of prescription items Aripiprazole	32	6536.12	326.46	5797.00	7117.00

Supplementary Table S3. DPS data per month over 32 months (Jan 2020-Aug 2022) for total costs (€), costs per example drug (€), number of patients per example drug and total number of prescription items, and number of prescription items per example drug.

Variable	Obs	Mean	Std. Dev.	Min	Max
Total Cost	32	€369154.13	43322.88	313912.50	462896.51
Total Cost Paliperidone	32	€38039.59	4199.23	28979.84	48260.63
Total Cost Quetiapine	32	€48950.07	7178.41	38879.74	65899.58
Total Cost Olanzapine	32	€32830.64	5139.53	26894.09	45455.76
Total Cost Aripiprazole	32	€35782.18	5091.99	28498.93	45867.46
No. of patients Paliperidone	32	79.44	10.28	52.00	96.00
No. of patients Quetiapine	32	2577.69	605.92	1495.00	3770.00
No. of patients Olanzapine	32	1460.03	306.96	888.50	2080.00
No. of patients Aripiprazole	32	543.50	131.00	320.50	816.00
Total No. of Prescription items	32	30637.44	4334.72	25698.00	40041.00
No. of prescription items Paliperidone	32	94.31	9.83	80.00	115.00
No .of prescription items Quetiapine	32	3229.81	610.73	2396.00	4555.00
No. of prescription items Olanzapine	32	1832.53	316.56	1473.00	2534.00
No. of prescription items Aripiprazole	32	672.72	136.41	526.00	988.00
